# A real-world analysis of patient-reported outcomes in patients with migraine by preventive treatment eligibility status in the US and Europe

**DOI:** 10.1186/s41687-020-00221-w

**Published:** 2020-07-06

**Authors:** Janet H. Ford, Shonda A. Foster, Russell M. Nichols, Antje Tockhorn-Heidenreich, Wenyu Ye, James Jackson, Sarah Cotton

**Affiliations:** 1 Eli Lilly and Company, 893 S. Delaware Street, Indianapolis, IN 46225 USA; 2 Eli Lilly and Company, Erl Wood Manor, UK; 3 Adelphi Real World, Bollington, UK

**Keywords:** Migraine, Patient-reported outcomes, Preventive eligibility, Burden of disease

## Abstract

**Background:**

Migraine has a severe impact on health-related quality of life (HRQoL) affecting physical, emotional, and social aspects of daily living of an individual. Preventive treatment has been demonstrated to improve HRQoL by reducing the frequency of migraine headache days.

**Methods:**

The study used data from 2017 Adelphi Migraine Disease Specific Program, which is a cross-sectional survey of physicians and their consulting patients with migraine in the United States (US) and five European countries (EU [Germany, France, UK, Italy and Spain]). Objectives were to evaluate patient-reported outcome (PRO) measures in the following two subgroups and by region (US and EU): (i) patients who are eligible for migraine preventive treatment (≥4 migraine headache days/month), and (ii) patients who are non-eligible for preventive treatment (< 4 migraine headache days/month). Patient-reported outcome measures that were assessed included the following: Migraine-Specific Quality-of-Life Questionnaire Version 2.1, Migraine Disability Assessment Scale (MIDAS), European Quality of Life-5 Dimensions-5 Levels version, and Work Productivity and Activity Impairment.

**Results:**

In total, 5462 patients (US = 1373; EU = 4089) were included in the study (preventive eligible: US = 584; EU = 1942; preventive non-eligible: US = 789; EU = 2147). In the US and EU, preventive eligible patients were significantly more likely to have worse disability as measured by MIDAS than non-eligible patients; preventive eligible patients also had significantly greater functional impairment, worse health utility, and overall greater work impairment (*p* < 0.0001). Among patients who were preventive eligible, a larger proportion of patients in the US reported that migraine forced them to reduce the number of hours worked as compared with the EU population (29.0% vs 24.7%).

**Conclusion:**

Patients who were preventive eligible (≥4 migraine headache days/month) demonstrated greater burden of disease across multiple PRO measures; trends were similar across the US and the five EU countries.

## Introduction

Migraine is a disabling neurological disease characterized by severe attacks of headache pain lasting 4 to 72 h accompanied by hypersensitivity to light and sound, nausea, vomiting, cognitive and vestibular symptoms [1]. Migraine affects > 10% of the adult population globally and is 2 to 3 times more common in women than in men [2, 3]. The prevalence of migraine is estimated to be 12% in the United States (US) and 15% in Europe (EU) [4–6]. The prevalence of migraine peaks during prime working ages (18**–**55 years old), thereby leading to substantial loss in productivity and high economic burden [7]. People with migraine experience diminished health-related quality of life (HRQoL) with wide-ranging adverse effects on physical, emotional, and social aspects of daily living including family, work, and social relationships [8, 9]. Among people with migraine, several factors contribute to the decline of HRQoL including symptoms of the disease, disease severity, and frequency of migraine attacks [10]. Even moderate migraine attacks can interfere with patients’ normal activities, and the loss of well-being occurs both during and between the migraine attacks [10, 11].

Treatment options include acute treatments at the time of migraine attack, and preventive treatments to reduce the number of migraine attacks. Treatment guidelines provide recommendations on when to initiate preventive treatment. Factors considered while recommending initiation of preventives include frequency of attacks (≥4 migraine headache days /month [preventive eligible]), decreased patient functioning despite acute treatment, issues with current acute treatment (e.g. contraindication, overuse, adverse events), and patient preference [12, 13]. Even though ~ 25% of people with migraine are estimated to be in need of a preventive therapy, notably fewer people with migraine are treated with a preventive drug despite the disabling nature of the disease [5, 14].

Prior research studies have defined subgroups by headache frequency, including chronic migraine (≥15 headaches per month of which 8 are migraine over a 3 month period), high-frequency episodic migraine (8**–**14 migraine headache days/month) and low-frequency episodic migraine (< 8 migraine headache days/month) [15]. Approximately 50% of patients with migraine are estimated to experience 0 to 3 migraine headache days/month and are typically not considered eligible for preventive treatment [5, 7]. However, preventive treatment is also considered among people with 2 to 3 migraine headache days/month who have moderate to severe impairments [14]. There exists a gap in research specific to the full migraine population, which is considered eligible for preventive treatment. Therefore, the primary objective of this study was to compare multiple patient-reported outcomes (PROs) using real-world data from the US and Europe among patients with migraine who are eligible for preventive treatment and who are not. Outcomes assessed included measures of patient functioning, disability, work productivity, and health utility.

### Study objectives

The primary objective of this study was to evaluate PROs related to the burden and impact of migraine within two subgroups, those defined as eligible for migraine preventive treatment (preventive eligible, > 4 migraine headache days/month) versus those defined as non-eligible for migraine preventive treatment (preventive non-eligible, < 4 migraine headache days/month). These were evaluated across the following regions: the US, EU and the total study population (US+EU). PROs included Migraine-Specific Quality of Life Questionnaire version 2.1 (MSQv2.1), Migraine Disability Assessment (MIDAS), European Quality of Life-5 dimensions-5 level version (EQ-5D-5L), and Work Productivity and Activity Impairment (WPAI) scores.

Secondary objectives included descriptive analyses of demographics and clinical characteristics for the two subgroups by region. Clinical characteristics included number of migraine headache days/month, average migraine severity over the last 3 months, presence of comorbidities and proportion of patients reported as being intolerant/refractory to their current preventive medication.

## Methods

### Study data and time period

This study used existing data from the 2017 Adelphi Migraine Disease Specific Program (DSP), which is a real-world, point-in-time, cross-sectional survey of primary care physicians, neurologists, and their consulting patients with migraine. Detailed information on the methodology used for DSPs has been published previously [16]. Briefly, the DSP consists of data pertaining to treatment practices, symptom prevalence, patient demographics, clinical outcomes, medication utilization, adherence patterns, productivity, and HRQoL. Data were collected from the US and five European countries (EU), including Germany, France, Italy, Spain, and the United Kingdom (UK). This survey was performed in full accordance with the US Health Insurance Portability and Accountability Act 1996, and each participant provided consent for de-identified and aggregated reporting of research findings.

The study data were collected cross-sectionally from August through December 2017. A total number of 152 physicians were recruited in the US (primary care physicians (PCPs):101; neurologists:51), 98 from France (PCPs:54; neurologists:44), 90 from the UK (PCPs:50; neurologists:40), 91 from Germany (PCPs:51; neurologists:40), 92 from Italy (PCPs:51; neurologists:41), and 92 from Spain (PCPs:52; neurologists:40).

Each physician completed a patient record form (PRF) for their next 9 consulting patients with a diagnosis of migraine; patients diagnosed with migraine could be attending the clinic for any reason. Patient demographics, history of diagnoses, details of migraine symptoms (e.g. number of migraine headache days, severity), comorbid conditions, treatment records (acute and preventive), and other disease management data were collected on the PRF. Patients were invited to complete a Patient Self-Completion (PSC) form; the PSC form collected patient-reported information including demographic details, treatment response and satisfaction, and validated PRO measures.

Inclusion criteria for this study were adult patients with migraine (≥ 18 years of age). PROs were specific to patients who completed the PSC form [17]. For the purposes of this research study, the definition of preventive eligibility was solely based on the migraine headache days frequency criteria from the treatment guideline recommendations of American Headache Society [17]. Patients meeting the frequency threshold of ≥4 migraine headache days/month were defined as preventive eligible. Migraine headache days were collected as the number on average, per month, over the last 3 months.

### PRO measures

MSQv2.1 is a self-administered health status instrument that measures the impact of migraine (over the last 4 weeks) on patients’ daily functioning. MSQv2.1 specifically addresses the impact of migraine on work or daily activities, relationships with family and friends, leisure time, productivity, concentration, energy, tiredness and feelings. The instrument consists of 14 items addressing three domain scores: Role Function Restrictive (RF-R), Role Function-Preventive (RF-P) and Emotional Function (EF). Raw scores are transformed onto a 0 to 100 scale, with higher scores indicating better daily functioning [18, 19]. The instrument is considered reliable, valid and sensitive to change in frequency of migraine attacks [18, 19].

MIDAS is a validated and reliable instrument that quantifies migraine-related disability over a 3-month period. The instrument consists of five items quantifying the number of days with missed work/school, missed household work, reduced productivity at work/school, reduced productivity in household work, and missed family or social activities. The total number of days for each item are added together to produce a total score with the following defined categories: little or no disability (0–5), mild disability (6–10), moderate disability (11–20), and severe disability (21+) [20–22].

EQ-5D is a multi-dimensional, HRQoL instrument that contains two components: a health status profile and a Visual Analog Scale (VAS). The health status profile allows patients to rate their health state on that day within five domains; higher score indicates a better health state as perceived by the patient [23]. In this study, country-specific tariffs were applied to generate health utility index scores, where 1.0 represents perfect health. VAS provides an overall patient rating of health status, with a range from 0 (worst imaginable health state) to 100 (best imaginable health state) [23].

WPAI questionnaire is a validated instrument that consists of four metrics measured over the past 7 days: absenteeism (work time missed), presenteeism (impairment while working), overall work productivity loss (overall work impairment) and impairment in daily activities (activity impairment). Scores are calculated as impairment percentages [24], with higher numbers indicating greater impairment and less productivity and therefore, worse outcomes [24].

### Statistical methods

Descriptive summary statistics (proportions for categorical variables and mean with standard deviation for continuous variables) were used to report the results among preventive eligible and preventive non-eligible subgroups. Continuous variables with an approximately normal distribution were compared using two-sample t-test. Continuous variables that were not normal and ordinal were compared using Mann-Whitney test. Categorical variables were compared using a Fischer’s exact test (small cell sample or binary outcome) or a Chi-square test. All statistical tests were performed at a two-sided 5% significance level (*p*-values < 0.05 was considered statistically significant). No adjustments for multiplicity were made for multiple comparisons. Sample size is less than the size reported in the demographic data due to non-responders or missing data, and no data were imputed (Stata version 15.1 was used to run the analysis).

## Results

### Patient disposition and demographics

In total, 5462 patients (US = 1373; EU = 4089) were included in the study after applying the eligibility criteria. The number of patients who were identified as preventive eligible and preventive non-eligible were as follows: US: preventive eligible: 584; preventive non-eligible: 789; EU: preventive eligible: 1942; preventive non-eligible: 2147; Total: preventive eligible: 2526; preventive non-eligible: 2936. Majority of patients were female and White (Table 1). Statistically significant differences between patients who were preventive eligible versus preventive non-eligible were observed for the following characteristics: for gender there were more females (US only), more were forced to reduce work hours due to migraine, and migraine severity was greater. Specific comorbidities such as depression and anxiety need to be considered while interpreting potential differences in PROs in both the regions. In both the US and the EU, depression was statistically significantly higher (*p* < 0.05) in preventive eligible patients compared with preventive non-eligible (Table 1).

**Table 1:** Baseline demographics and migraine disease characteristics by preventive treatment eligibility status

Demographics	US		EU^**a**^			Total (US + EU)
	Preventive Eligible^**b**^	Preventive Non- Eligible^**c**^	Preventive Eligible^**b**^	Preventive Non- Eligible^**c**^	Preventive Eligible^**b**^	Preventive Non- Eligible^**c**^
	n = 584	*n* = 789	*n* = 1942	*n* = 2147	*n* = 2526	*n* = 2936
Age (years), mean (SD)	41.3 (14.2)	41.1 (14.6)	39.7 (13.4)	39.7 (14.4)	40.1 (13.6)	40.1 (14.5)
Female, %	77.6	72.5*	69.3	70.6	71.2	71.1
White/Caucasian, %	77.6	73.5	90.6	91.8	87.6	86.9
Patient employment status (%)*						
Working full time	55.8	62.2	51.3	51.8	52.4	54.6
Working part time	9.8	11.4	10.9	10.0	10.6	10.4
Retired	6.3	6.1	6.7	7.6	6.6	7.2
Unemployed	4.3	1.6	5.7	5.8	5.4	4.7
On long-term sick leave	1.9	0.4	1.8	0.7	1.8	0.6
Number of migraine headache days/month (%) ^†^						
0–3	0	100.0	0	100.0	0	100.0
4–7	59.2	0	70.8	0	68.1	0
8–14	27.2	0	22.6	0	23.6	0
15+	13.5	0	6.7	0	8.3	0
Concomitant conditions, %						
Depression	21.9	17.0*	11.9	9.7*	14.3	11.6**
Anxiety	27.9	22.9*	20.2	18.7	22.0	19.9
Stress	15.8	15.3	14.1	15.1	14.5	15.2
Migraine condition ever forced them to reduce hours worked	*n* = 383	*n* = 581	*n* = 1209	*n* = 1327	*n* = 1592	*n* = 1908
Yes (%)**	29.0	17.2	24.7	19.5	25.8	18.8
Average migraine severity over the last 3 months,	*n* = 575	*n* = 783	*n* = 1922	*n* = 2117	*n* = 2497	*n* = 2900
Mean (SD) (1 = very mild, 10 = severe) ^†^	6.4 (2.1)	5.2 (2.1)	6.1 (1.7)	5.2 (2.0)	6.2 (1.8)	5.2 (2.0)
Patient refractory to preventive medication	*n* = 305	*n* = 271	*n* = 810	*n* = 640	*n* = 1115	*n* = 911
Yes (%)**^d^	8.2	1.5	9.3	3.6	9.0	3.0
Current preventive therapy, % (most frequent)	n = 584	*n* = 788	*n* = 1941	n = 2147	*n* = 2525	*n* = 2935
No preventive drug treatment	49.0	66.8^†^	59.1	71.1^†^	56.8	69.9^†^
Anticonvulsants	26.7	14.7^†^	10.6	7.3**	14.3	9.3^†^
Antidepressants/ anxiolytics/ benzodiazepines	12.8	6.3^†^	9.5	5.9^†^	10.3	6.0^†^
Beta blockers	10.8	10.2	15.1	11.3**	14.1	11.0**

^†^*p* < 0.0001; **p < 0.01; *p < 0.05 for comparisons between preventive eligible and preventive non-eligible

^a^European (EU) countries included: Germany, France, Spain, Italy and, the UK

^b^Preventive Eligible was defined as > 4 migraine headache days/month. ^c^Preventive Non-Eligible was defined as > 4 migraine headache days/month

^d^Patients who are currently receiving a preventive treatment

Abbreviations: *SD* Standard deviation

### PRO measures

**Fig. 1:**
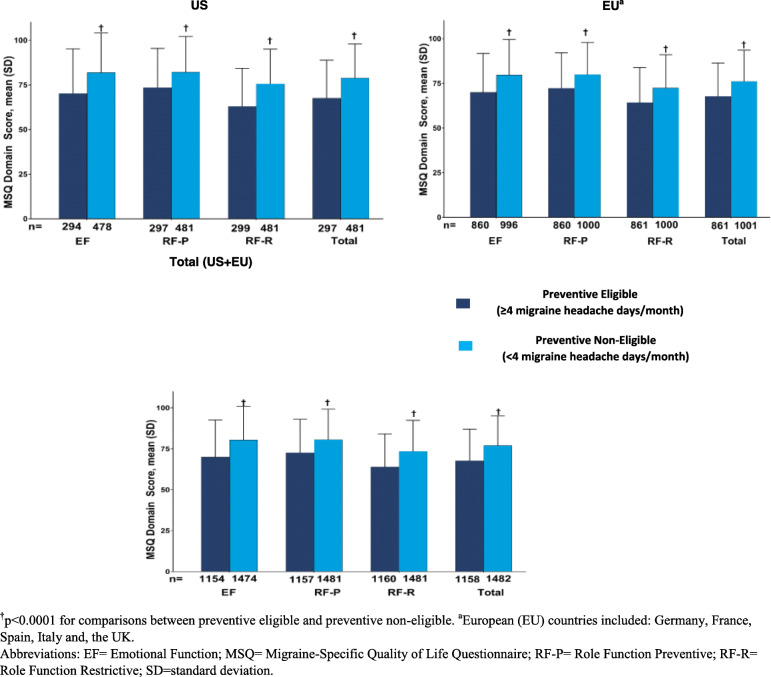
MSQ domain score, mean (SD) in patients with migraine by preventive treatment eligibility status

MSQ mean [SD] total scores were statistically significantly lower in patients who were preventive eligible compared with those who were preventive non-eligible, indicating greater functional impairment in this population (US: 67.6 [21.2] vs 78.8 [19.1]; EU: 67.7 [18.6] vs 76.1 [17.4]; Total: 67.7 [19.3)] vs 77.0 [18.0]; *p* < 0.0001). Individual item scores also demonstrated a similar trend (Fig. 1).

**Fig. 2:**
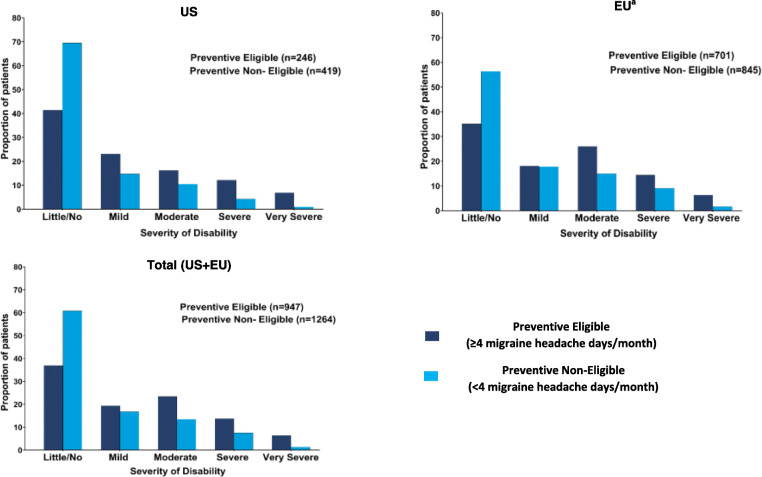
Migraine disability grade per the MIDAS (%) by preventive treatment eligibility status

For MIDAS, preventive eligible patients were significantly more likely to have worse disability than non-eligible patients in both the US and EU (*p* < 0.0001) (Fig. 2). The proportion of preventive eligible patients who were experiencing moderate or severe disability was higher in the EU (26.0% and 14.4%, respectively) compared with the US population (16.3% and 12.2, respectively). Mean scores for individual items of MIDAS are summarized in Table 2.

**Table 2:** Patient reported migraine disability individual item scores (mean [SD]) by preventive treatment eligibility status

MIDAS individual items, mean (SD)	US		EU^**a**^			Total (US + EU)
	Preventive Eligible^**b**^ (n = 584)	Preventive Non- Eligible^**c**†^ (n = 789)	Preventive Eligible^**b**^ (n = 1942)	Preventive Non- Eligible^**c**†^ (n = 2147)	Preventive Eligible^**b**^ (n = 2526)	Preventive Non- Eligible^**c**†^ (n = 2936)
**Number of days missed work or school**	1.5 (2.4)	0.7 (1.5)	1.7 (4.6)	0.7 (1.5)	1.7 (4.1)	0.7 (1.5)
**Number of days with reduced productivity by half or more at work or school**	2.9 (7.0)	1.0 (1.9)	2.8 (5.4)	1.5 (3.0)	2.8 (5.9)	1.4 (2.7)
**Number of days missed of household work**	4.6 (9.5)	1.6 (4.3)	4.0 (7.1)	1.9 (3.0)	4.2 (7.8)	1.8 (3.5)
**Number of days with reduced productivity by half or more in household work**	4.7 (9.2)	1.5 (3.1)	4.2 (7.0)	2.3 (3.7)	4.3 (7.6)	2.0 (3.6)
**Number of days missed family or social events**	2.2 (3.3)	1.1 (2.2)	3.0 (4.1)	1.6 (2.5)	2.8 (3.9)	1.4 (2.4)

^†^*p* < 0.0001 for comparisons between preventive eligible and preventive non-eligible

^a^European (EU) countries included: Germany, France, Spain, Italy and, the UK

^b^Preventive Eligible was defined as > 4 migraine headache days per month. ^c^Preventive Non-Eligible was defined as < 4 migraine headache days/month

Preventive eligibility groups derived from PRF data; MIDAS is a patient reported outcome tool included in the patient self-completion form and so individual bases are lower

Abbreviations: *MIDAS* Migraine-Disability Assessment Score, *SD* Standard deviation

**Fig. 3:**
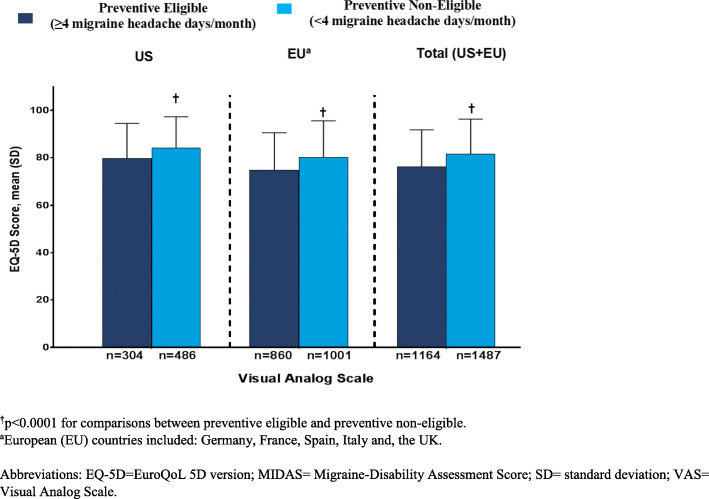
EQ-5D VAS score in patients with migraine by preventive treatment eligibility status

**Fig. 4:**
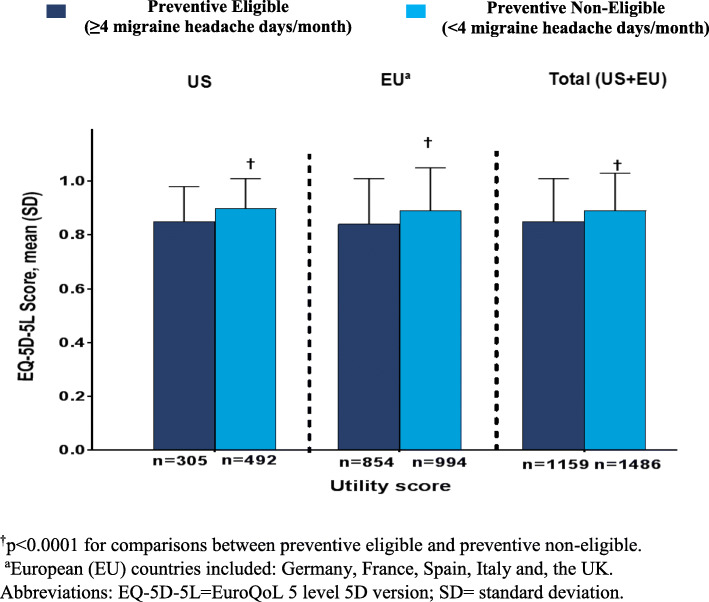
EQ-5D-5L utility score in patients with migraine by preventive treatment eligibility status

EQ-5D-5L scores indicated worse health state and lower health utility among patients who were preventive eligible (Fig. 3 and Fig. 4). Health utility scores (mean [SD]) were significantly lower among patients who were preventive eligible compared with those who were preventive non-eligible in the EU (0.84 [0.17] vs 0.89 [0.16]) and total population (0.85 [0.16] vs 0.89 [0.14]) (*p* < 0.0001); however, similar scores were observed in the US population (0.85 [0.13] vs 0.90 [0.11]). Mean VAS (mean [SD]) scores were statistically significantly lower among patients who were preventive eligible compared with those who were preventive non-eligible (US: 79.8 [14.7] vs 84.3 [13.0]; EU: 74.8 [15.8] vs 80.2 [15.4]; Total: 76.1 [15.7] vs 81.5 [14.8]; *p* < 0.0001).

**Fig. 5:**
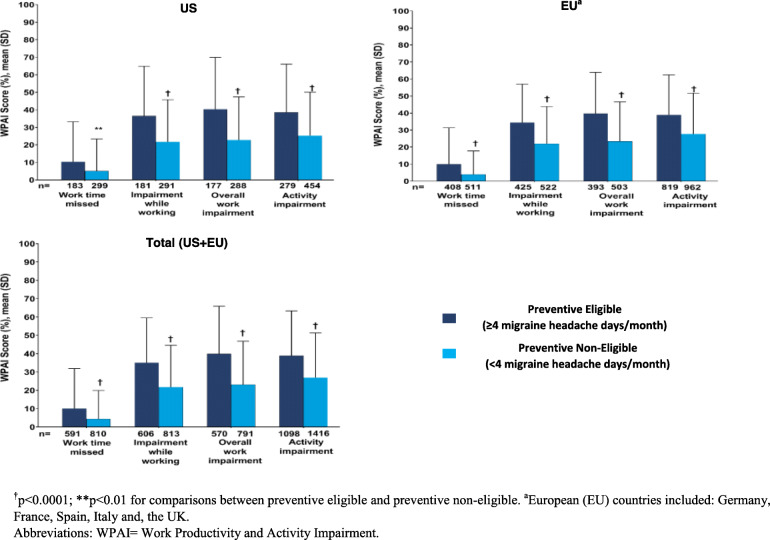
WPAI score (%) in patients with migraine by preventive treatment eligibility status

Mean percentage of overall work impairment due to migraine was statistically significantly higher among patients who were preventive eligible compared with those who were preventive non-eligible across the regions (US: 40.3% vs 22.7%; EU: 39.7% vs 23.4%; Total: 39.9% vs 23.1%, respectively; *p* < 0.0001). Similar trends were observed for other WPAI scores including percentage of work time missed, impairment while working and activity impairment, due to migraine (Fig. 5).

## Discussion

The current study findings suggest that, across the US and EU, trends are consistent with greater patient burden due to migraine among preventive eligible patients (defined as > 4 migraine headache days/month) compared with those who are preventive non-eligible (defined as < 4 migraine headache days/month). MSQ scores indicated greater functional impairment with domain and total scores being statistically significantly lower among preventive eligible compared with non-eligible patients. Similarly, preventive eligible patients were significantly more likely to have worse disability than non-eligible patients, as per the MIDAS scores. EQ-5D-5L scores reflected worse health status per VAS ratings, and lower health utility among patients who were preventive eligible versus those who were not. Furthermore, mean percentage of overall work impairment due to migraine was statistically significantly higher among patients who were preventive eligible as compared with those who are non-eligible.

The contributions of this study are important, as previously published literature has majorly focused on the burden of episodic migraine versus chronic migraine [25, 26]; however, the current study specifically addressed the migraine population that would be considered eligible for preventive treatment based on the treatment guidelines [12, 13]. This research contributes a deeper clinical understanding of the patient population that needs to be considered for preventive treatment as compared with the population for whom only acute migraine treatment is appropriate. This is a key factor considered by health care providers and healthcare policy makers in decision-making.

Previous studies have addressed HRQoL measures associated with migraine across multiple countries [27]. The Eurolight study conducted across multiple European countries showed that migraine has a significant impact on personal relationships, work, household, and social activities of individuals [28]. The American Migraine Prevalence and Prevention study, and other international studies have also shown that burden of migraine increases with frequency of migraine attacks [25, 29]. Our study focused on patient functioning and migraine-related disability in the US and EU. Our study results are consistent with the Eurolight study, with significant impact of migraine on relationships and missed work, as per MSQ and WPAI scores. The results suggest that greater proportion of patients who were preventive eligible have higher levels of disability ranging from moderate to very severe (MIDAS) and lower functioning (MSQ) compared with preventive non-eligible patients. This demonstrates that headache-related disability increases with increase in frequency of migraine headache days/month across both the US and EU. These results are consistent with previously published studies that showed the relationship between greater disability and lower MSQ scores with increase in frequency of migraine headache days (≥15 migraine headache days/month) [25, 26]. Similar association was observed among patients with migraine with ≤3 migraine headache days/month versus patients in higher frequency subgroups (4–14 migraine headache days/month) in the U.S [7].

In relation to work productivity, this study demonstrated significantly higher levels of impairment (~ 2-fold increase in overall work impairment) in patients who were preventive eligible compared with preventive non-eligible patients. Previous studies have demonstrated reduced work productivity with greater headache frequencies [27, 28]. In addition, the present study showed lower health utility scores, and health status ratings in the higher frequency subgroup (> 4 migraine headache days/month), which is consistent with previous studies [30]***.***

### Strengths/limitations

The strengths of this study included that data were collected in real-world settings across multiple countries via the same methodology enabling cross-country comparisons. All patients had a physician confirmed diagnosis of migraine, and both health-care provider reported data and PROs were collected. Limitations include that study participation was optional introducing the potential for selection bias, where non-participating sites and respective patients may have differed from those represented in this study. Specifically, physicians who were approached to participate in this study routinely see a large volume of patients and are experienced with treating migraine and were more likely to be from urban areas. Patient participant rates for the PSC form were 49% and the characteristics of participants versus non-participants were similar; notable differences were that participants had been diagnosed with migraine for a longer duration, were relatively less likely to be eligible for preventive treatment, and relatively more likely to receive prescription treatment. The sample is representative of the consulting population of patients with migraine; however, the results may not be generalizable to the wider migraine population such as those in rural areas and who are undiagnosed or have less severe illness. In addition, for the purpose of this study, patients with 2 to 3 migraine headache days/month were considered as preventive non-eligible. If degree of impairment is considered in addition to frequency, a subset of these patients (< 25%) would have met preventive eligibility status per MIDAS criteria for moderate to severe disability. Another limitation is the cross-sectional design of the study; causality cannot be prospectively assessed and there is a potential for recall bias. Furthermore, the objectives and results of this study are descriptive in nature with unadjusted analyses; therefore, the findings may alter when adjusting for demographic imbalances between the groups. Notably, observed differences between the two subgroups included the percentage of patients with depression or anxiety, and sex (US only); these may have contributed to observed differences in PROs. However, this study was designed to specifically reflect real-world patient outcomes in clinical settings without controlling for other variables. This research does address important research questions specific to patients who are considered eligible for preventive migraine treatment, with an evaluation across multiple PROs and geographies.

## Conclusion

In conclusion, among patients with migraine who are considered eligible for preventive treatment, there is a high unmet need as consistently demonstrated by various HRQoL measures, with similar trends across multiple countries (US and EU). Among these patients who are considered preventive eligible per treatment guidelines, initiating a preventive treatment may lead to substantial improvements in migraine headache days, and has the potential to decrease the burden of illness associated with migraine.

## Data Availability

The data that support the findings of this study are available from Adelphi Real World, but restrictions apply to the availability of these data, which were used under license for the current study and so are not publicly available. However, data are available from the authors upon reasonable request and with permission from Adelphi Real World.

## Abbreviations

DSP: Disease-specific program
EQ-5D-5L: European Quality of Life-5 Dimension-5 Level
EF: Emotional Function
EU: European Union
HRQoL: Health-related quality of life
MIDAS: Migraine Disability Assessment Scale
MSQ: Migraine-Specific Quality-of-Life Questionnaire
PCPs: Primary care physicians
PRF: Patient record form
PROs: Patient-reported outcomes
PSC: Patient self-completion form
RF-P: Role Function Preventive
RF-R: Role Function Restrictive
SD: Standard deviation
UK: United Kingdom
US: United States
VAS: Visual Analog Scale
WPAI: Work Productivity and Activity Impairment
